# Sustainability practices and organizational performance during the COVID-19 pandemic and economic crisis: A case of apparel and textile industry in Sri Lanka

**DOI:** 10.1371/journal.pone.0288179

**Published:** 2023-07-11

**Authors:** Naween Weerasinghe, Ashani Weerasinghe, Yulashika Perera, Sanduni Tennakoon, Nilmini Rathnayake, Punmadara Jayasinghe

**Affiliations:** SLIIT Business School, Sri Lanka Institute of Information Technology, Malabe, Sri Lanka; National Textile University, PAKISTAN

## Abstract

The apparel and textile industry is the backbone of the Sri Lankan economy, contributing significantly to the country’s gross domestic product (GDP). The coronavirus (COVID-19) pandemic, which also triggered the ongoing economic crisis in Sri Lanka, has a profound effect on the organizational performance of apparel sector firms in Sri Lanka. In this context, the study examines the impact of multi-dimensional corporate sustainability practices on organizational performance in the said sector. The study employed the partial least squares structural equation modelling (PLS-SEM) technique for analysing and testing the hypothesis of the study while using Smart PLS 4.0 software as the analysis tool. Relevant data were collected through a questionnaire from 300 apparel firms registered with the Board of Investment of Sri Lanka (BOI). The study results indicated that "economic vigour,” “ethical practices," and "social equity" have a significant impact on organizational performance, while "corporate governance" and "environmental performance" have an insignificant impact. Unique discoveries from this study would be useful to prosper organizational performance and formulate novel sustainable future strategies not limited to the garment industry even during harsh economic conditions.

## Introduction

The apparel and textile industry is the backbone of the Sri Lankan economy, generating the highest export revenue as an industry and a key contributor to the country’s GDP. According to an online interview review, the garment sector contributes to over 50% of exports while providing over 350,000 direct and 700,000 indirect job opportunities through their supply chain activities [1]. Also, according to the statistics provided by the Export Development Board (EDB), the industry has exported over USD 3,4 million worth of garments in 2019, mostly to key buyers such as the United States of America, European Union and Middle Eastern countries, Japan, and Australia [2]. This explained the significance of garment exports, which was one of the major reasons for selecting the apparel industry as the base of the current study. Other than that, previous experience of the authors gained by working within the industry and the pandemic’s significant impact resulting up to a 40% decline in performance, also led to selecting the apparel industry [2].

Sri Lankan export sector has been adversely impacted by the harsh economic conditions caused by a series of incidents beginning with the Easter Sunday bombing in 2019. Within a year, the coronavirus (COVID-19) outbreak has declared a pandemic that pushed the local governmental authorities to impose sudden lockdowns island wide as preventive health measures. This scenario worsened the unfavourable economic conditions Sri Lanka experienced, thereby dwindling production rates almost in all industries [3]. In other words, the health crisis developed into an economic crisis, which was much felt with no signs of recovery, especially for developing nations like Sri Lanka relying on a few exports like apparel. Lockdowns on major dollar-value-generating industries, such as apparel manufacturing and tourism, had a significant impact, resulting in an economic crisis with United States Dollar (USD) shortage [4]. By 2022, Sri Lanka’s economic downturn exerted the country’s inflation rate to be among the highest in Asia [5]. As evidenced by the EDB statistics, the COVID-19 pandemic condition and the current economic crisis have significantly impacted the organizational performance of the Sri Lankan apparel and textile industry, indicating a massive decline in export dollar revenue under both circumstances between 2020 and 2021. This staggering decline was despite an estimated steady increase of at least USD 2.7 billion in the garment industry [2]. Reasonably, the prevailing economic conditions compelled organizations to put a higher focus on survival-based strategies rather than sustainability-based strategies. In this setting, the present study made an effort to highlight the importance of corporate social responsibility (CSR) even during tough economic conditions.

Gradually, CSR advanced from a concept to a reality with the growing interest of stakeholders. Therefore, it is essential for most corporates to demonstrate their contribution to society and ethical standards [6]. Generally, CSR is defined as a three-dimensional or "triple-bottom-line" with a combination of social, economic, and environmental dimensions [7], whereas some studies defined CSR as a combination of multiple dimensions [8]. Nowadays, most firms commonly practice CSR in line with increasing customer expectations, as a tool for handling competition in business [9]. Further, CSR positively impacts corporate image, where public perceives the company as a responsible entity in society. Strong and positive public perception builds company’s customer loyalty and improves public perception [10]. Besides these, CSR practices positively impact brand awareness, image, satisfaction, and loyalty while increasing the perceived product quality [11]. This questions whether sustainability practices can positively affect Sri Lanka’s apparel industry even during the pandemic and economic crisis situations.

Organizations formulate sustainability strategies, thus, vary from firm to firm [12]. But these are prone to fail if non-aligned with the organization’s business strategies. Typically, firms take a narrow focus on shareholders, whereas, on the overall, sustainability broadly focused on stakeholders [13]. This shows that managers must adopt innovative and up-to-date sustainability strategies [14], since the apparel and textile industry is one of the most polluting industries and severely damages the ecological environment [15]. However, rich literature cannot be found regarding the multi-dimensional view of sustainability practices beyond the triple bottom line. Most studies have been conducted within the context of developed countries, and limited knowledge is available from the developing countries’ perspectives [16, 17]. Notably, there is a scarcity of knowledge on sustainability practices in this subject, which affected the sustainability performance in developing countries like Sri Lanka. Therefore, this present study is critical because it fills the research gap in terms of a lack of comprehensive sustainability practices covering multiple dimensions of sustainability beyond the triple bottom line. In doing so, the study investigates how multiple sustainability practices impact the organizational performance of the apparel and textile industry firms from a developing country perspective. Comprehending the situation can contribute to mitigate the impact of the current crisis on the apparel industry’s performance.

Firstly, during the investigation, a thorough understanding of corporate sustainability practices, its effect on organizational performance and limitations under challenging conditions are discussed. These can add valuable inputs to strengthen existing policies to dampen the impact on this sector during this turbulent period. Secondly, to expand an emerging concept called sustainability, two more dimensions, ethics and corporate governance, are added as new knowledge. In a period of recession, how organizational performance is affected by sustainability practices requires management leadership and guidance to optimize decision making for survival of the Sri Lankan apparel firms supplying for global brands; it can also attract more investments to this sector. Thirdly, as the concept of "sustainability” originated in the context of developed countries, the existing literature suffers from a lack of knowledge on the context of developing countries and a lack of empirical research limited to mere theoretical justifications. The present study’s empirical evidence enriches the existing literature on this subject from developing countries’ perspectives. Finally, so far, a few research studies have been conducted in the Sri Lankan sustainability context by applying the PLS-SEM analysis. Therefore, this is the initial study where the survey data was analysed and presented as a model using the PLS-SEM, the findings can be generalised to other developing countries.

### Key problem statement

Sustainability is an emerging concept the world is willing to embrace but still it is at the evolutionary stage where gaps remain. Obviously, now-a-days, sustainability is more than environmental conservation driving the concept towards the triple bottom line approach, also considering the economic and social aspects [16]. But it is clear that sustainability concept is stuck in a common framework without comprehensive sustainability practices in place, hence not adequately covering all the aspects of sustainability even beyond the triple bottom line. It is because focusing only on social, environment, and economic dimensions are insufficient to explain the concept of sustainability. According to [18], it is also where most scholars turn a blind eye. The situation is similar in the business world. When managers make decisions only based on the triple bottom line approach, the organizational performance tend to be curtailed because unexplained dimensions exist beyond the triple bottom line that often go unnoticed [16]. Therefore, to generate a better organizational performance, it is essential that decision makers investigate the multiple dimensions beyond the triple bottom line [19].

Other than that, the literature cannot be regarded as rich, considering the impact of sustainability practices on organizational performance in the context of Sri Lankan apparel industry and developing country perspective, along with prior empirical studies [16]. The apparel industry is considered one of the most polluting industries in the world. Contradictorily, it is also considered to be the backbone of economies like in Sri Lanka, Therefore, disregarding the industry is not an option, whereas sustainability practices that are multi-dimensional in nature, are essential to generate a better sustainability performance from such industries.

Overall, the problem statement was generated as lack of comprehensive set of sustainability practices covering multiple dimensions of sustainability beyond the triple bottom line during the COVID-19 and the economic crisis in the context of Sri Lanka’s apparel industry. Following the problem statement, the study intended to bridge the gap between the known and unknown, while shedding light on the grey area beyond the triple bottom line. In order to fill the gap, the study followed a multidimensional framework of sustainability covering five dimensions of sustainability including ethical, and corporate governance aspects. The study raises five questions, one for each dimension (1) what is the impact of economic vigour on organizational performance during COVID-19 and economic crisis situation? (2) what is the impact of environmental performance on organizational performance during COVID-19 and economic crisis situation? (3) what is the impact of social equity on organizational performance during the COVID-19 and economic crisis situation? (4) what is the impact of corporate governance on organizational performance during COVID-19 and economic crisis situation? (5) what is the impact of ethical practices on organizational performance during COVID-19 and economic crisis situation?

To address the above-mentioned questions in the current study, the following five specific objectives are identified as below:

1. To investigate the impact of economic vigour on organizational performance during COVID-19 and economic crisis situation
2. To investigate the impact of environmental performance on organizational performance during COVID-19 and economic crisis situation
3. To investigate the impact of social equity on organizational performance during COVID-19 and economic crisis situation
4. To investigate the impact of corporate governance on organizational performance during COVID-19 and economic crisis situation
5. To investigate the impact of ethical practices on organizational performance during COVID-19 and economic crisis situation

By understanding the impact generated by each dimension, it is expected to explain the importance of multi-dimensional view of sustainability even during the tough economic conditions. As such. identifying the critical dimensions that require much attention of the management and in resource allocation, is a pressing priority with the economic downfall.

The paper layout includes the following components: Section 1 provides an introduction, and Second 2 reviews the literature and the underlying concepts related to variables discussed in this study. Section 3 discusses data and methodology after that. The study results are discussed in Section 4, and Section 5 discusses the results. Section 6 provides a brief conclusion of the research. Finally, section 7 highlights limitations and future research directions respectively.

## Literature review

Most research studies have investigated the triple bottom line approach of sustainability limiting to the environment, social, and economic aspects [16]. When managers make decisions based on the triple bottom line concept, the organization’s performance tend to be low, mainly due to lacking consideration on other aspects of sustainability. According to [18], triple bottom line failed to fully capture the notion of sustainability, resulting in a gap regarding the dimensions beyond the triple bottom line. Hence, no longer triple bottom line can reflect the boost in organizational performance. Therefore, to generate a better performance, it is essential to consider the overall dimensions, focusing on the wider picture beyond the triple bottom line. [20] stated that the ESG framework also does not adequately cover the sustainability concept; within the limitations, the authors suggested to use intangible measures to assess organizational performance and for path analysis in future studies. Hence, as previously mentioned, the study’s problem statement is the lack of a comprehensive set of sustainability practices covering multiple dimensions than the triple bottom line approach to support decision makers, to generate better performance in the apparel industry of Sri Lanka even during pandemic and economic crisis. Supporting the above statement, [21] mentioned that the impact of triple bottom line can change with time and the situation in which CSR practices have been implemented, therefore, considering multi-dimensional aspects is essential mostly for decision makers.

With that, in order to identify the prevailing knowledge level, Wiley Online Library, ResearchGate, ScienceDirect, Emerald, and Google Scholar databases were accessed to develop the literature review, while corporate sustainability, the triple bottom line, the Sri Lankan apparel industry, dimensions of sustainability practices, economic vigour, social equity, corporate governance, environmental performance, and organizational performance were used as keywords when searching for published research articles. Further, a metric is used to assess the effects of sustainability practices on organizational performance, considering economic vitality, environmental performance, social equity, corporate governance, and ethical practices as the independent variables. (S1 Fig) indicates the article selection process [18].

### Economic vitality

Organizations in Sri Lanka have been severely hit by both COVID-19 and the economic crisis concurrently. Moreover, economic vitality is a critical component of sustainability. According to prior studies, achieving sustainable growth is challenging while avoiding environmental destruction [22]. On top of this, the ability to generate long-term income largely depends on competitive value strategies and economic sustainability considerations [17, 23]. Economic sustainability practices mainly include alternative energy, sustainable agriculture, cryptocurrencies, blockchain technology, recycling, pollution reduction, and sustainable fisheries [24]. Sustainability related issues are encountered by the apparel and textile industry in the supply chains. But blockchain technology can ensure sustainable supply chain excellence by lowering human errors, detecting unethical suppliers, minimising cost of supply chain failures and transactional times. In contrast, technological and system related barriers, human resource and R&D barriers hinder the adoption of blockchain in the industry [25].

Previous studies conducted within the context of Sri Lanka’s apparel industry concluded that effective CSR practices positively impact economic performance [22] and boost long-term shareholder value [26]. However, some studies focused on corporate sustainability regarding profitability [27]. Although the industry generates an encouraging impact on the economy, it imposes negative impacts on the environment through resource depletion, pollution, and greenhouse gas emissions [28]. Considering the economic and social point of view, most research studies investigated only employee welfare, cost analysis, and employee safety as indicators within the scope of the study but suggested that comprehensive indicators are required when assessing the sustainability of the apparel and textile industry [28]. According to another study, the environmental impact of the Sri Lankan apparel sector is greater than that of the United States (US) due to differences in electricity generation, but the economic benefits for workers and worker safety are much higher than most US counterparts. As per the above-mentioned study, although apparel manufacturing in the US has better benefits in terms of environmental sustainability, Sri Lankan apparel manufacturing has shown positive effects in terms of economic sustainability but with adverse environmental consequences. Thus, the study emphasized the importance of considering multi-dimensional sustainability [18].

The citations above clearly mention that organizational performance and economic vigour are directly and indirectly essential for developing nations like Sri Lanka. Additionally, studies state that economic vitality significantly influences organizational performance [29]. In addition, economic vigour was contemplated as a crucial component that should be investigated within the apparel and textile industry throughout the period under study (i.e., COVID-19 and economic crisis situations). Referring to these literary works in the present investigation, the first hypothesis is constructed as follows,

**Hypothesis 1:** Economic vigour significantly impacts organizational performance

### Environmental performance

Environmental sustainability means satisfying the requirements of both current and future generations without endangering the ecological system’s well-being [30], In other words, it refers to the protection of the ecological environment. Most (94%) of previous studies focused on environmental sustainability individually or with other dimensions in the context of manufacturing firms [31], illustrating the importance of environmental concerns. According to [32], despite common goals in all contexts, the methods of achieving environmental sustainability can differ according to situational conditions, and the main reason is that developed-country strategies may not be applicable to developing-countries, which are sector-specific. A study validated that ISO certification, waste material ratio, water consumption, wastewater ratio, pollution and renewable energy ratio had been identified as the sustainable ecological production indicators in the apparel and the textile industry [33]. As per another study, risk related to social and environmental aspects require high priority due to the high probability of exposure to media channels indicating the importance of environmental sustainability [34]. However, as the long-term profit aspect is often associated with green investments, some firms targeting short-term profits tend to ignore the concept of environmental sustainability [35].

[36] disclosed that environmental performance could impact an organization’s financial performance, encouraging organizations to use sustainable business models to integrate the green practices with business strategies [37]. In order integrate sustainability strategies successfully, knowledge management plays a major role. However, still managerial barriers, innovation, and technological barriers can generate deviations [38]. Although it is essential, environmental sustainability practices are increasingly being used to improve financial performance in developed countries [39] than in developing countries. Moreover, [40] revealed that firms that adopt innovative environmental practices tend to gain a competitive advantage over other market participants, illustrating the importance of implementing green practices. Before the financial crisis, a strong positive relationship was evident between environmental performance and financial performance among listed firms in the Australian context [41]. Apart from these, an insignificant association between the respective variables has been found via an empirical analysis [42] and [43] argued that extremely diversified measurements had been used to measure corporate environmental performance, hence it is impractical to identify a reliable measurement.

The association between environmental factors and firm performance has been widely tested and found to have a significantly positive impact, regardless of varying impacts in different contexts [16]. But still a knowledge gap exists in the literature regarding the impact of environmental sustainability practices on organizational performance due to a lack of studies in the apparel industry’s context in developing countries [44]. Besides, the unfavourable impact aggravated in COVID-19 pandemic times and the economic crisis within the apparel and textile industry in Sri Lanka. In light of the above findings, the second hypothesis for the study can be formed as below,

**Hypothesis 2:** Environmental performance significantly impacts organizational performance

### Social equity

Sustainability provides a simple lens through which many conflicting demands of modern society may be witnessed [45] and social equity states that every member deserves to be treated with dignity and have equal rights to participate in society through the three main classifications of corporate social sustainability, sustainability proactiveness, sustainability management, and sustainability bargaining [46]. Social sustainability became crucial with the rise of unethical violations of social norms with industrial development [47]. Social persistence, working conditions, health and safety, connectedness with employees, human rights, wellness, diversity, fair labour practices, charity, and community involvement are a few instances of how and to what extent a firm has successfully translated its social goals [48]. Despite these claims, sustainability components may differ from business to business.

Prior studies have applied social sustainability primarily based on the function(s) of one specific company [49]. Here; the scholars declared that CSR policies are detrimental to corporate shareholders due to their high cost of implementation and other related reasons. Considering the textile and garment industry with labour-intensive outsourcing tactics in developing countries notorious for high levels of corruption draws attention to the social components [28]. Certain studies suggested conceptual frameworks for social sustainability [50]. In addition, some studies were developed to evaluate societal sustainability employing a series of methodological conundrums framed by four fundamental traits, which led to the creation of social factor research studies in the Sri Lankan context. These studies evaluated the significance of managing corporate social responsibility in light of various stakeholders, and methods for assessing social sustainability have been developed and used in research projects across the globe [51]. In contrast to positive correlations in several studies, negative or very weak correlations were also found between sustainability variables and organizational performance [48]. However, since fewer studies are available on the topic, the third hypothesis was generated to measure the impact of the pandemic and economic crisis.

**Hypothesis 3:** Social equity significantly impacts organizational performance

### Corporate governance

The set of factors that influence management’s decision making and controlling of an organizational body is known as CG, and corporations exercise a mixture of tough and soft laws for control purposes [52]. The alignment of stakeholder interests and governance caused a rise in the topic of CG, influencing the focus of scholars. [53] emphasized that CG skills and knowledge are required to ensure the long-term improvement of shareholder value while balancing other stakeholder interests and reducing the risk of environmental damage. Embracing the sustainability concept and embedding it in overall operations and resources, as well as continuous monitoring, is essential to achieving it [54]. A trend of active engagement of stakeholders towards the topic can be witnessed in modern industries improving the importance of CG [55].

Even though a few studies focus on the relationship between CSR and CG, some provide a modern conceptual framework of CG practices [56] and literature reviews of previous studies [53]. Previous research abundantly highlighted the impact of leadership maintenance, execution, formation, and sustainability in the context of multi-dimensional aspects of CSR, thus raising suggestions to measure and understand leader motivation for CSR [57]. Active community engagement and transparency are required to ensure good governance [58], and internal and external audits of corporate practices impact the corporation’s long-term viability [59]. [60] formed a framework connecting CG practices and their effects on the environment and community systems [60]. Noticeably, mechanisms considered in previous studies for improving stakeholder involvement can also be used by corporations [61]. Internal and external governance systems are essential for sustainability [62], and it was evident that CG significantly impacted strategic planning [63].

Regardless of a dearth of studies from developing countries’ perspectives, available findings emphasize diverse impacts [64, 65]. According to recent research, combined ESG performance score as well as individual social and governance score have a significant positive impact on firm value and profitability [66]. However, as mentioned previously (under hypothesis one), the study variable can be impacted by the economic setback in the period under study in Sri Lanka, where a research gap exists in the apparel industry domain. With the expectation of testing the gap, the fourth hypothesis was generated as follows,

**Hypothesis 4:** Corporate governance significantly impacts organizational performance

### Ethical practices

Organizations frequently engage with society and people; ethical and social movements began in the 1960s and became a vital discipline within management [67]. Before the evolution of ethics, some domains not governed by the law were later addressed by ethical practices, in which the court system does not interfere in violation of ethics, while society solely imposes consequences. Governance procedures, market manipulation, corruption, bias, corporate accountability, fiduciary duty etc., is mostly known as business ethics. While laws usually do not address business ethics, business ethics occasionally provides a fundamental norm that firms may adhere to win public approval [68].

Past research studies have defined business ethics as right and wrong or good and bad human behaviour in a corporate environment [69]. Moreover, these scholars conclude that ethical practices foster trust among customers, businesses, and other market participants [70] while balancing the legality of business activities to ensure a long-term economic advantage over the competition. The existing literature evidence a positive relationship between business ethics and CSR [71]. A quantitative study concluded that ethical practices impact organizational performance when senior management of a firm generates performance strategies in harmony with strong ethical practices [72]. Another study supports the positive impact, concluding that organizations must treat ethical considerations critically to avoid future risks associated with court cases and negative business reputations [73]. Another study found that unethical business standards have no positive impact on organizational performance while emphasizing the importance of incorporating ethical practices into business operations [74]. Although most research studies support the argument that business ethics positively impact organizational performance, some reach contradictory conclusions, implying a weak relationship or an impact from ethical practices [75].

A code of ethics is established with the aid of long-term ethical principles by incorporating the straightforward behaviour of all workers, from the highest levels of management to the newest and the youngest, therefore, the success of organizations is dependent on business ethics. This means that when all workers act ethically, the firm gains reputation and other advantages [76], which is tested from the fifth hypothesis,

**Hypothesis 5:** Ethical practices significantly impact organizational performance

### Organizational performance

In research on management disciplines, most researchers used organizational performance as the dependent variable. Nevertheless, definition of the concept is still open, as only a few studies have evidenced its definitions and measures. Early studies identified organizational performance as a multidimensional construct [77, 78], while a comparative study proved that both subjective and objective measures are equivalent. Opposingly, [79] suggested the possibility of using that subjective measures to effectively gauge the firm performance, generally due to management’s reluctance to disclose objective figures.

Most research studies on sustainability and firm performance have been conducted in developed countries. Moreover, the study results depend on the cultural and economic aspects, indicating no universal impact between sustainability and firm performance [80]. Due to low generalizability, most researchers have commonly used financial indicators as an alternative to measure firm performance [81, 82]. Practitioners seek financial benefits from sustainability as outcomes of a strategy, while policymakers expect to make the industry more sustainable, concerning policy implications. A scholar found that sustainability strategies impact sustainable innovation using PLS-SEM for the analysis, where such innovations positively impact overall firm performance [83]. The impact of sustainability on firm performance was recently investigated in India using PLS-SEM, as evidenced from a developing country perspective [84]. During the COVID-19 pandemic, eco-product development has had an insignificant impact on SME performance due to low productivity in lockdown situations, generating unfavourable effects on the above-mentioned relationship [85]. Generally, the relationship between corporate sustainability and organizational performance has been discovered to have a major positive influence on firm performance. However, in addition to having a considerable positive influence, it also has various impacts in different circumstances [16]. However, the magnitude of the impact may intensify during the said period regarding Sri Lanka’s garment and textile industries. It is because the apparel industry is engaged in supplying to international markets, and experiences both global and local crisis situations simultaneously [86]. As per the above findings, most past studies have assumed organizational performance as the dependent variable. Hence, this study’s dependent variable is also stated as organizational performance.

**Fig 1:**
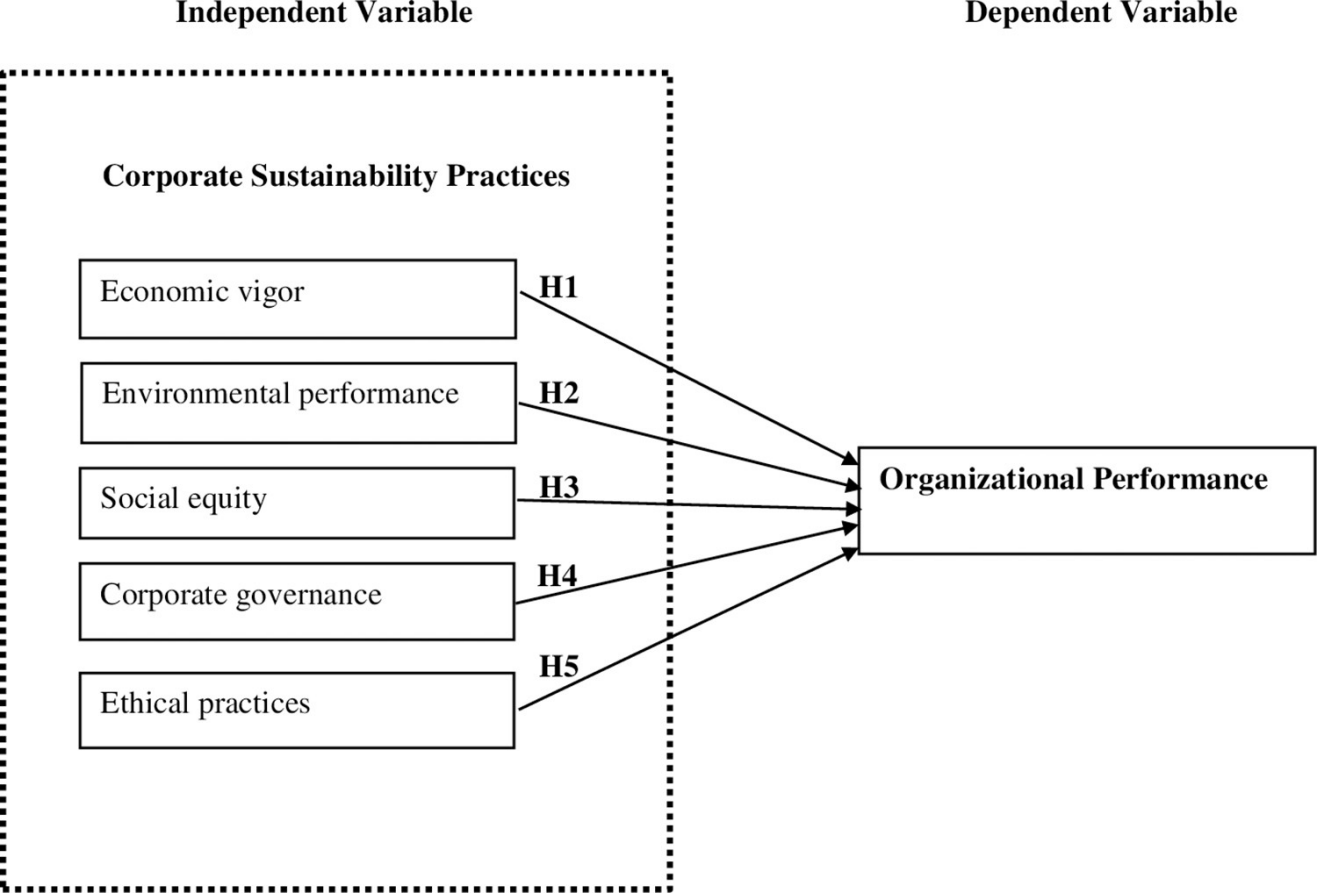
Conceptual framework of the study. Source: Author’s compilation based on literature review.

Based on the above-stated hypotheses, the authors developed the following conceptual framework for the study (Fig 1).

## Methodology

The research design procedure, involving data gathering, data analysis procedures and methods, are summarized in S2 Fig.

Data was gathered utilizing a close-ended questionnaire to quantify the study’s latent variables and predetermined answers were provided for respondents to select from. Each latent variable is measured using indicators represented by sustainable practices. Table 1 shows which sustainable practices were taken into account for this study. Before answering the online survey, study participants verbally agreed to participate. However, they were offered the opportunity to withdraw at any time. The data was acquired anonymously, and no privileges were provided to any participant in exchange for their involvement in this research. Consequently, all participants in this study—researchers and respondents—declared that they have no conflict of interest connected to the current study.

**Table 1:** Sustainability practices under consideration of the study.

**Economic Sustainability Practices**	
EC1	The company is using the latest manufacturing techniques.
EC2	The company is investing in research and development to increase the company revenue through innovations.
EC3	The company is using electronic media to buy and sell.
EC4	The company is entering new markets.
EC5	The company is recycling to reclaim materials, or remanufacturing and repacking returned goods.
**Environmental Sustainability Practices**	
EN1	The company is applying Life Cycle Analysis in the product designing process.
EN2	The company is using renewable energy sources (Solar panel).
EN3	The company is applying lean manufacturing.
EN4	The company has an Effluent Treatment Plant to recycle, and reuse wastewater.
EN5	The company is evaluating the vendor’s environmental performance and select an eco-friendly vendor.
EN6	The company is participating in collaborative planning sessions with vendors to address issues related to the environment.
EN7	The company is utilizing technology for transportation and route optimization.
EN8	The company is using ecologically friendly packaging (Ex: Low-density packaging, and biodegradable packaging).
**Social Sustainability Practices**	
SO1	The company is providing a training and personal development process for employees.
SO2	The company is considering employee working conditions, health, and safety.
SO3	The company is offering healthcare coverage for employees.
SO4	The company is offering job opportunities for disabled employees.
SO5	The company has an action plan to improve stakeholder involvement.
**Corporate Governance Sustainability Practices**	
CG1	The company has an independent representation of directors.
CG2	The company has environmental leadership.
CG3	The company has a board-level sustainability committee.
CG4	The company has a practice of transparency.
CG5	The company is using corporate governance education.
**Ethical Sustainability Practices**	
ET1	The company has a code of ethics.
ET2	The company has a system in place to safeguard whistle-blowers.
ET3	The company has a strategic plan to drive an organization toward a more ethical culture.
ET4	The company has an ethics training committee and ethics trainings for employees.
ET5	The company is evaluating the role of organizational ethics on the rest of sustainability aspects.
**Organization Performances**	
OP1	There is an increase in net income after implementing above sustainability practices during the pandemic and economic crisis.
OP2	There is a decrease in employee turnover rate after implementing above sustainability practices during the pandemic and economic crisis.
OP3	There is an increase in number of new products developed after implementing above sustainability practices during the pandemic and economic crisis.
OP4	There is an increase in the number of sponsorships after implementing sustainability practices during the pandemic and economic crisis.
OP5	There is an increase in the number of training sessions after implementing sustainability practices during the pandemic and economic crisis.

Source: Authors’ compilation

The study population comprised apparel and textile enterprises registered with the Board of Investment of Sri Lanka (BOI), which conducted business throughout the pandemic and economic crisis. Currently, the BOI list of registered apparel companies is regarded the most updated, thus, was the main reason to select it. According to the BOI list, 306 apparel and textile enterprises are registered in Sri Lanka at the time of the study, which constitute the study’s population. According to the Krejcie and Morgan chart, the sample size for analysis was determined as 169 companies among the overall population [87]. The current study attempted to cover the entire population to generate a better validity and reliability aiming for generalizability for the entire apparel sector. However, only 300 responses (with a response rate of 98.04%) were collected from the population.

The company’s participation was entirely voluntary and primarily linked through its connections within the BOI and the Biyagama Export Processing Zone. To measure the applicability of the questionnaire, a pilot survey was conducted by randomly selecting 16 companies from the study sample before sharing the survey in September 2022; thereafter, the finalized questionnaire was sent out to the entire population in October 2022. The collection process spanned from September to December 2022, and thereafter, the analysis was conducted considering a single apparel company in Sri Lanka as the unit of analysis. Completing the data collection process in December 2022, 300 responses were collected under the simple random sampling method from the population of 306 apparel companies selected from the list of companies registered with the BOI. The questionnaire was distributed through emails; some were filled through phone conversations with a response rate of 98.03%, receiving 300 responses that underwent the analysis process. In terms of survey respondents, 22.6% were general managers, 14.8% were sustainability managers, and the remaining 62.6% employees were represented by other positions in the selected 300 companies according to Table 2.

**Table 2:** Demographic information of the respondents.

Demographics	Categories	Percentage (%)
**Years of Operation**	1–10	16.3
	10–20	18.9
	20–30	36.3
	30–40	19.3
	40–50	9.3
**Size of the Company**	SME	41.1
	Large	58.9
**Respondent Position**	General Manager	22.6
	Manager Sustainability	14.8
	Other	62.6
**Type of the Company**	Sole Proprietor	14.1
	Partnership	0
	PVT Limited Company	85.6
	Public Limited Company	0.4

Source: Authors’ compilation

The collected dataset was analysed using PLS-SEM to assess the five hypotheses of the study. It attracted more attention from scholars than covariance-based structural equation modelling (CB-SEM). After the dominance of CB-SEM in 2010, most researchers used the PLS-SEM method because of its ability to estimate complex models with a multitude of variables and the availability of user-friendly software like Smart PLS and PLS-Graphs, which only require simple technical knowledge to conduct analysis [88]. Additionally, the SEM method allows researchers to conduct factor and path analyses simultaneously using the same software for analysing complex models [89]. Due to its affordability and ability for free testing by new users, the Smart PLS version 4.0 software was chosen for the analysis procedure. Following the two-way approach, the measurement and the structural models were assessed accordingly to generate the study’s results [90]. Due to the above-explained reasons, most research studies in the period 2014–2022 used PLS-SEM as the analysing mechanism. This is similar to methodological backgrounds, which also validated the selection of PLS-SEM to assess the data set [8, 91, 92].

## Results

### Descriptive statistics

The first variable, CG, has five main indicators coded as (CG1), (CG2), (CG3), (CG4), and (CG5). The range of all the mean values is at an average level, between the lowest value (3.217) and the highest value (3.457). The standard deviation values of CG are in the range of 1.038 and 1.097. The second variable economic vigour consists of five indicators coded as (EC1), (EC2), (EC3), (EC4), and (EC5). Table 1 presents the separate mean values in the range of 3.360 and 3.583, while the standard deviation is between 1.066 (lowest) and 1.131(highest), as shown by Table 1. The environmental performance variable consists of eight indicators coded as (EN1), (EN2), (EN3), (EN4), (EN5), (EN6), (EN7), and (EN8). The mean values are between 3.303 and 3.853, while the standard deviation values are between 1.020 and 1.177. The latent variable ethical practices include five indicators coded as (ET1), (ET2), (ET3), (ET4), and (ET5); its mean values disperse between (3.337) and (3.497) while the standard deviation values are between (1.058) and (1.167). The social equity latent variable includes five variables coded as (SO1), (SO2), (SO3), (SO4), and (SO5); its mean values spread from (3.263) to (3.313) while the standard deviation is between (1.114) and (1.133). The study’s dependent variable organizational performance consists of five indicators coded as (OP1), (OP2), (OP3), (OP4), and (OP5); its mean values are spread between (3.200) and (3.463) while the standard deviation is between (1.085) and (1.206) Table 3.

**Table 3:** Descriptive statistics of main independent and dependent variables.

Indicator	Mean	Median	Min	Max	Standard Deviation
CG1	3.217	3.000	1.000	5.000	1.085
CG2	3.293	4.000	1.000	5.000	1.077
CG3	3.337	3.000	1.000	5.000	1.038
CG4	3.457	4.000	1.000	5.000	1.090
CG5	3.287	3.000	1.000	5.000	1.097
EC1	3.433	4.000	1.000	5.000	1.131
EC2	3.493	4.000	1.000	5.000	1.094
EC3	3.410	4.000	1.000	5.000	1.093
EC4	3.583	4.000	1.000	5.000	1.066
EC5	3.360	4.000	0.000	5.000	1.115
EN1	3.853	4.000	1.000	5.000	1.122
EN2	3.810	4.000	1.000	5.000	1.020
EN3	3.757	4.000	1.000	5.000	1.060
EN4	3.683	4.000	1.000	5.000	1.109
EN5	3.717	4.000	1.000	5.000	1.100
EN6	3.687	4.000	1.000	5.000	1.150
EN7	3.303	3.000	1.000	5.000	1.025
EN8	3.433	4.000	1.000	5.000	1.177
ET1	3.393	4.000	1.000	5.000	1.058
ET2	3.337	4.000	1.000	5.000	1.106
ET3	3.487	4.000	1.000	5.000	1.109
ET4	3.497	4.000	1.000	5.000	1.112
ET5	3.383	4.000	1.000	5.000	1.167
OP1	3.463	4.000	1.000	5.000	1.138
OP2	3.200	3.000	1.000	5.000	1.206
OP3	3.253	3.000	1.000	5.000	1.164
OP4	3.390	4.000	1.000	5.000	1.148
OP5	3.303	4.000	1.000	5.000	1.085
SO1	3.287	3.000	1.000	5.000	1.124
SO2	3.313	3.000	1.000	5.000	1.120
SO3	3.263	3.000	1.000	5.000	1.120
SO4	3.297	4.000	1.000	5.000	1.114
SO5	3.287	3.000	1.000	5.000	1.133

Source: Authors’ compilation based on Smart PLS output

Note: CG = Corporate Governance Practices, EC = Economical Practices, EN = Environmental practices, ET = ethical practices, OP = Organizational performance, SO = Social Sustainably practices.

Regarding average mean scores, the indicator (EN1) has the highest effect on organizational performance, while the indicator (CG1) has the least. Regarding the standard deviation, the indicator (EN8) shows the highest spread while the indicator (EN2) shows the lowest. Both mean and standard deviation values of variables indicators represent its dispersion, hence, the importance of independent variables.

### Measurement model results

The adequacy of the model was examined and reported by assessing the reliability, convergent validity, and discriminant validity in line with the selected criteria of Cronbach’s alpha value, AVE statistics, and HTMT ratio.

#### Reliability statistics

Cronbach’s Alpha is a commonly used statistic to confirm whether the scale is acceptable for the study. It is one of the most essential and comprehensive statistics in research connected to analyzing the study’s consistency and reliability of the dataset. Cronbach’s alpha value should be more than 0.7 to establish the research’s reliability [93]. If any independent variable fails to score this value, it implies that the variable is unreliable and does not have internal consistency.

According to Table 4, the latent independent variable CG has an alpha value of (0.915) higher than 0.7, which falls within the criteria that makes the variable reliable. Similarly, all the other independent variables can be considered reliable for the study, as alpha values for economic vigour, environmental performance, ethical practices, and social equity are 0.894, 0.890, 0.912 and 0.931, respectively, which are higher than the value as per the above-mentioned criteria. The dependent variable, organizational performance, indicates an alpha value of (0.931) satisfying the criteria; therefore, it is considered reliable for the study. It can be concluded from the statistics that the latent variables are reliable and have internal consistency to proceed with the study.

**Table 4:** Cronbach’s alpha values of main study variables.

Latent Variable	Cronbach’s Alpha Value
Corporate Governance	0.915
Economic Vigour	0.894
Environment Performance	0.890
Ethical Practices	0.912
Organizational Performance	0.931
Social Equity	0.931

Source: Authors’ compilation based on Smart PLS output

#### AVE statistics

Validity can be described as how well the scale can measure, i.e., what it is intended to measure to be categorized into two parts, i.e., convergent and discriminant validity. Convergent validity can be defined as how well each item is converged to represent its underlying construct. Convergent validity can be established via the Average Variance Extracted (AVE) score, which means how much variance is extracted by the latent constructs based on the indicators. The minimum requirement of the AVE score should be greater than (0.50) to reach an acceptable level [93]. The output of the Smart PLS results generated the AVE scores of each variable as corporate governance (0.743), economic vigour (0.704), environment performance (0.548), ethical practices (0.740), organizational performance (0.785), and social equity variable as (0.784). Hence, the scores of each latent variable showed a greater value than (0.5), demonstrating convergent validity as per Table 5.

**Table 5:** AVE values.

Latent Variable	Average Variance Extracted
Corporate Governance	0.743
Economic Vigour	0.704
Environment Performance	0.548
Ethical Practices	0.740
Organizational Performance	0.785
Social Equity	0.784

Source: Authors’ compilation based on Smart PLS output

#### Discriminant validity

The discriminant validity was used to evaluate how well the tested constructs varied from the other constructs [94]. Mainly three methods could be utilized to analyse the discriminant validity: Fornell-Larcker Criterion, Heterotrait-Monotrait (HTMT) ratio, and Cross Loadings. HTMT is a novel criterion for measuring discriminant validity in variance-based SEM that estimates the correlation between the components. [95] have critically argued that the Fornell-Larcker criteria and cross-loading evaluation have an unacceptably low sensitivity, implying that these are often incapable of identifying a lack of discriminant validity. According to these scholars, the HTMT value should be less than 0.9 when subjected to a reflective model, and if this value is more than this threshold, there is a lack of discriminant validity. All HTMT values in Table 6 are less than 0.9, demonstrating the discriminant validity of constructs.

**Table 6:** HTMT ratio of latent variables.

	CG	EC	EN	ET	OP	SO
**CG**						
**EC**	0.327					
**EN**	0.099	0.512				
**ET**	0.214	0.797	0.484			
**OP**	0.252	0.728	0.396	0.755		
**SO**	0.223	0.805	0.486	0.779	0.685	

Source: Authors’ compilation based on Smart PLS output

Note: CG = Corporate Governance Practices, EC = Economical Practices, EN = Environmental practices, ET = ethical practices, OP = Organizational performance, SO = Social Sustainably practices

Before inventing the HTMT method, the scholars used the Fornell-Lacker Criterion to measure the discriminant validity of a model [95] for over 30 years. However, with time, addressing the limitations identified, a new method was invented. According to the criteria, AVE square root value of each latent variable must be greater than the correlations between other latent variables. Therefore, the diagonal values should be higher than all the respective vertical and horizontal values of the model as shown in Table 7.

**Table 7:** Fornell-Laker criterion values of latent variables.

	CG	EC	EN	ET	OP	SO
CG	**0.862**					
EC	0.308	**0.839**				
EN	0.093	0.498	**0.740**			
ET	0.207	0.722	0.482	**0.860**		
OP	0.245	0.668	0.422	0.697	**0.886**	
SO	0.220	0.737	0.502	0.718	0.641	**0.885**

Source: Authors’ compilation based on Smart PLS output

Note: CG = Corporate Governance Practices, EC = Economical Practices, EN = Environmental practices, ET = ethical practices, OP = Organizational performance, SO = Social Sustainably practices

### Structural model results

The coefficient of determination (R2) was used to assess the predictive capacity of the suggested model, whereas the coefficient of determination reveals that the complete variation in the dependent variable occurred because of an independent or exogenous variable. This analysis explained 0.556 variations in organizational performance, demonstrating that all corporate sustainability practices considered in this study contribute to a 56% variation in organizational performance as per Table 8.

**Table 8:** R square values.

R-square	R-square adjusted
0.556	0.549

Source: Authors’ compilation based on Smart PLS output

To identify the significance of the impact between the latent variables, the authors tested the structural model of the survey by bootstrapping, which consider a nonparametric procedure. Bootstrapping was used to compute the beta value, t-statistic, and p-value of the link between the five independent variables and organizational performance under the significant level of (0.05). The t-statistics value greater than (1.96) and the p-value less than (0.05) mean an independent variable significantly impacts the dependent variable. Results are summarized in Table 9.

**Table 9:** Path coefficient values.

Path	Beta Value	T-Statistic	P-Value
Corporate Governance → Organizational Performance	0.053	1.183	0.237
Economic Vigour → Organizational Performance	0.242	3.523	0.000
Environment Performance → Organizational Performance	0.032	0.656	0.512
Ethical Practices → Organizational Performance	0.379	6.529	0.000
Social Equity → Organizational Performance	0.162	2.452	0.014

Source: Authors’ compilation based on Smart PLS output

H1 proposed that economic practices are significantly related to organizational performance. There is a significant positive impact between economic vigour and organizational performance based on the t-statistic value (3.523), the p-value (0.000), and the beta value (β = 0.242). Therefore, H1 is accepted, which illustrates the better the economic practices performed in the organization, the better will be organizational performance. H2 proposed that environmental practices are significantly related to organizational performance. The t-statistic (0.656), as well as the p-value (0.512) and the beta value of (β = 0.032), indicate that environmental performance has an insignificant positive impact on organizational performance; hence H2 is unsupported. H3 proposed that social sustainability practices are significantly related to organisational performance. It can be inferred from the results that social sustainability practices significantly positively impact organizational performance (β = 0.162, p = 0.014, t = 2.452); thus, H3 is accepted. This illustrates the better the social practices performed in the organization, the better will be organizational performance. H4 proposed that CG practices are significantly related to organizational performance. Table 5 indicates the t-statistic (1.183), p-value (0.237), and beta value (β = 0.053), ensuring the positive insignificant impact between corporate governance and organizational performance. Hence H4 is rejected. H5 proposed that ethical practices are significantly related to organizational performance. When considering the relationship between ethical practices and organizational performance, the results show a t-statistic value of (6.529), a p-value of (0.000), and a beta value (β = 0.379), which means ethical practices have a significant positive impact on organizational performance. H5 is accepted and reflects that if a firm strongly adopts ethical practices, the corporate image from the perspective of various stakeholders like customers, suppliers, and distributors is strong, ultimately impacting their organizational performance.

As a reflective model, the values on the structural model are the outer loading values (Table 10) reflect how the latent variable influence on the corresponding indicator.

**Table 10:** Outer loading values.

	Outer loadings
CG1 <- CG	0.910
CG2 <- CG	0.890
CG3 <- CG	0.905
CG4 <- CG	0.802
CG5 <- CG	0.796
EC1 <- EC	0.867
EC2 <- EC	0.871
EC3 <- EC	0.837
EC4 <- EC	0.825
EC5 <- EC	0.791
EN1 <- EN	0.766
EN2 <- EN	0.760
EN3 <- EN	0.695
EN4 <- EN	0.754
EN5 <- EN	0.785
EN6 <- EN	0.751
EN7 <- EN	0.687
EN8 <- EN	0.717
ET1 <- ET	0.847
ET2 <- ET	0.895
ET3 <- ET	0.860
ET4 <- ET	0.848
ET5 <- ET	0.849
OP1 <- OP	0.847
OP2 <- OP	0.878
OP3 <- OP	0.910
OP4 <- OP	0.912
OP5 <- OP	0.882
SO1 <- SO	0.892
SO2 <- SO	0.904
SO3 <- SO	0.899
SO4 <- SO	0.866
SO5 <- SO	0.865

Source: Authors’ compilation based on Smart PLS output

**Fig 2:**
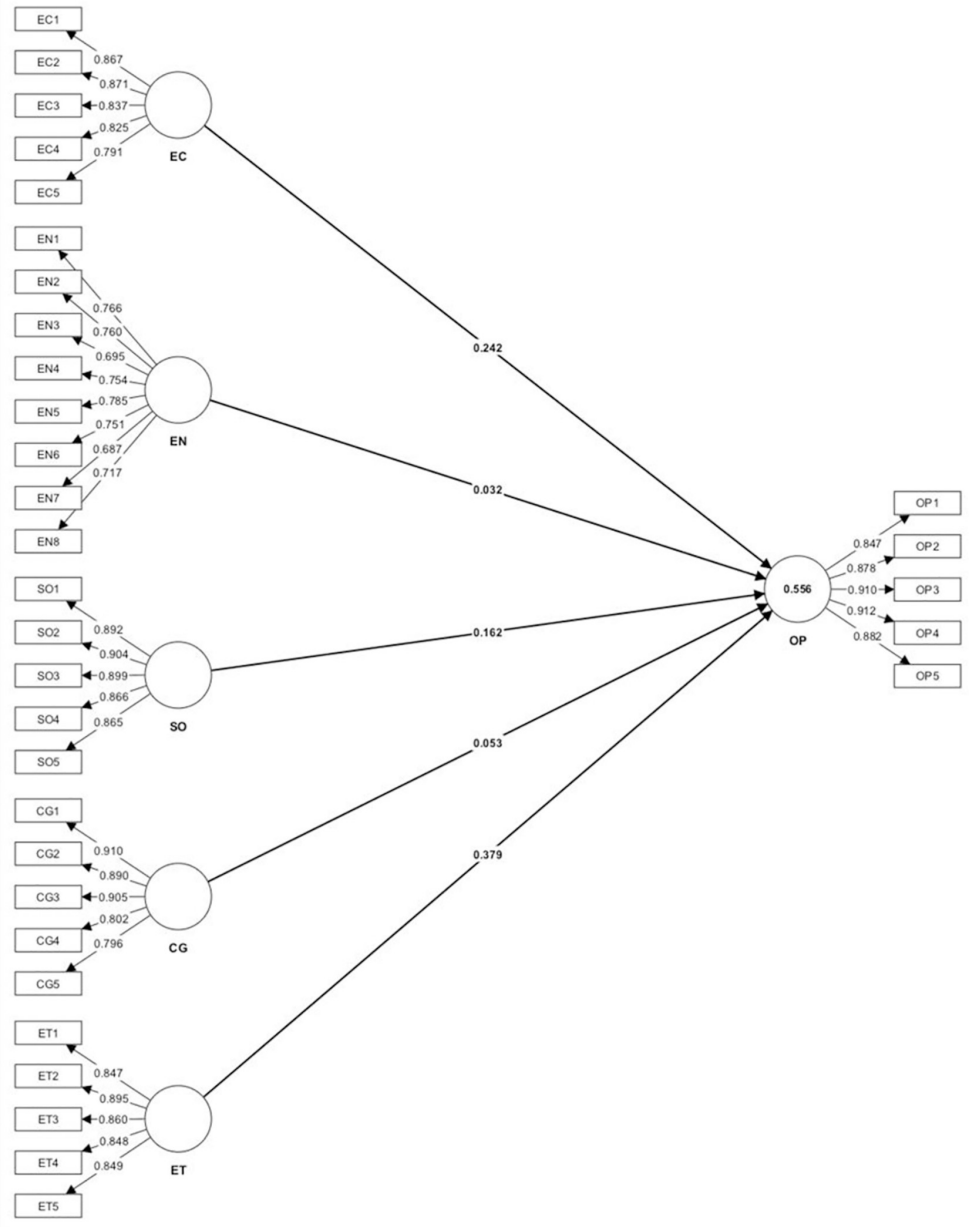
Smart PLS results of the structural model. Source: Authors generated from Smart PLS.

Accordingly, among these five independent variables, CG and environmental performance have an insignificant impact on organisational performance, whereas economic vigour, ethical practices, and social equity have a significant impact on organisational performance even during the COVID-19 and the subsequent economic crisis, which is the period under study. The structure model of the analysis process generated through Smart PLS software is presented in Fig 2.

## Discussion

The current study was carried out to determine which sustainability strategies substantially influenced organizational performance during the two crises (COVID-19 and the subsequent economic crisis) in the context of the Sri Lankan apparel industry. Further, this industry is a major polluter in the Sri Lankan economy. While addressing the corporate sustainability concept through five dimensions, the study results showed that economic sustainability practices, social sustainability practices, and ethical sustainability strategies have a substantial influence on the organizational performance of Sri Lankan apparel industry firms, while remaining practices, such as environmental practices and CG practices, do not do so.

The results indicated that the latent variable ethical practices significantly impact organizational performance. The path coefficient test with a p-value of (0.000) is lower than the alpha value of the study (0.05). As such, the respective hypothesis (H5) can be accepted. The result was also supported within the Chinese context, which evidenced a significant impact [8]. Additionally, past studies on different aspects of ethical practices significantly supported the findings in Cameroon [71] and the US contexts [96]. Similar results were found where ethical and cultural practices positively impacted competitive performance within the Chinese and Pakistan contexts [97]. Affirming these findings, a positive relationship was found between ethical leadership practices, justice, and trust as aspects of ethical sustainability practices and financial performance, highlighting the importance of ethical practices in improving organizational performance within the Korean context [98]. The SMEs in the South African context display unethical practices generating weak performance throughout business operations, indicating the significance of ethical practices to achieve better organizational performance. Furthermore, a similar study recommended treating ethical matters critically to avoid the risk of generating costs through negative brand reputation and court cases [73]. Assuming the past study findings are similar from the developed and developing country perspectives, the Sri Lankan apparel industry must also consider ethical practices and moral obligations to achieve better organizational performance even during the above-explained crises.

Similarly, in the present study, the latent variable of social equity positively impacts organizational performance in the Sri Lankan context. It generates a p-value of (0.014) lower than the acceptance criteria, the alpha value (0.05); thus, the third hypothesis can be accepted. Past research concludes that a positive relationship exists between social capital and organizational performance within the Turkish context since organizations are built around people and social communities [91]. Social capital indicates the linkages or the connections between the associations and people, including all external and internal stakeholders of the business. The said variable is a key aspect of the social sustainability dimension, and consideration towards each association can improve organizational performance. For example, strong bonds with employees lead to synergy effects, while a strong bond with investors leads to stable financial capabilities [99]. Astoundingly, the strength of the culture as an aspect of social sustainability has no relationship with organizational performance, contrary to social beliefs on the significance of strong culture [100]. As explained before, past research studies also affirm the significance of social practices; organizations engage with people and associations; thus, the impact is similar for Sri Lankan organizations.

Like the previously explained two latent variables, economic practices positively impact organizational performance. The path coefficient test indicates a p-value of (0.000) lower than the alpha value of the study (0.05), accepting the hypothesis (H1). Lesser past research studies exist regarding economic practices than other latent variables; however, economic sustainability practices mainly include alternative energy, sustainable agriculture, cryptocurrencies, recycling, pollution reduction, and sustainable fisheries [24]. In contrast, previous research studies show a negative relationship between alternative energy practices and organizational performance. Furthermore, the blockchain and cryptocurrency systems allow companies to gain new ways of minimizing costs and time to improve organizational performance [101]. In addition, recycling practices have a significant positive impact on organizational performance through new sources of income and cost savings in various aspects [102]. Despite the availability of studies for individual practices illustrating the significance of economic practices towards organizational performance, studies focusing on combined dimensions are lacking to support the present study’s investigation.

The latent variable environmental practices have an insignificant positive impact on organizational performance. The path coefficient indicates a p-value of (0.512); hence, hypothesis (H2) is rejected. [103] discovered in research conducted in a transition economy setting that higher environmental performance enhances profitability by driving down costs more than it drives down revenues. South Africa-based research [92] discovered that environmental sustainability strategies contribute favourably to innovation and small firms’ ecological and social performance. The study also discovered that environmental management techniques are negatively associated with their market and financial success [104]. Only limited researchers have studied the association between these two variables; nonetheless, the same constructs were investigated and discovered [105]. Here, the analysis revealed insufficient evidence for improving environmental performance. This is because ISO 14001 implementation leads to improved business performance, which may be seen as one factor required for corporate sustainability. The conclusion confirmed the findings indicating insignificant results in a meta-analysis of 19 papers on the association between environmental performance and financial success [106]. Lately, findings proved how environmental and social practices of CSR are related to the economic, social, and environmental performance of Mozambique firms from the managers’ perspective. According to the study, the influence is positive, although insignificant, validating the findings of this study [107].

The latent variable CG practices have an insignificant impact on organizational performance during the COVID-19 pandemic and economic crisis. It generated a p-value of (0.237) higher than the acceptance criteria and the alpha value (of 0.05). Since the variable is higher than the acceptance rate, the study illustrates that significant data is unavailable to accept the fourth hypothesis. Surprisingly, previous studies capture the above-explained significant impact and a strong positive relationship between CG and organizational performance. a strong relationship exists with the board size, CG mechanisms, communication strategies, and the code of conduct in terms of CG aspects, disagreeing with the current results study. Hence, even in the Sri Lankan context, strong CG practices and policies can be recommended to critically consider the avoidance of corporate collapses for improved organizational performance [108]. Another Sri Lankan study evidenced that good CG practices lead to better organizational performance considering the leadership structure, board composition, and board committees [109]. However, the current study result is supported by previous studies conducted within the Indian context considering the registered companies. According to the study, CG is not currently capable of having a major influence on organizational performance [110]. A significant impact existed before the COVID-19 pandemic and the subsequent economic crisis of the country as opposed to studies after the above-mentioned period, which shows an insignificant impact in this regard. Therefore, the study can conclude that CG practices have been critically affected by the pandemic and the country’s economic crisis.

As per the study results, the impact of environmental practices and CG practices on the performance of the apparel companies is insignificant. A similar study in the Indian context also revealed an insignificant impact between corporate sustainability performance in terms of ESG and corporate financial performance. It is because most firms are seen to be adopting sustainability practices for moralistic perspectives than strategic perspectives [20]. Additionally, global textile waste is significantly increasing, threatening the environmental sustainability, of which, 25% is recycled and reused where the rest is landfilled. As the waste is not recycled, it is not utilized for production of biogas and fiber, which no longer enhance the apparel firms’ performance [111]. On the other hand, condemning CSR activities [112] also pushes back apparel firms’ commitments towards sustainability. Some ideologies pinpoint that those companies charging high prices to gather funds form their CSR activities, though seemed to have embraced the CSR concept and publicising the same few CSR activities carried out before over and over again [113] and [114]. Moreover, consumption patterns are significantly changing with the global economic crisis [115]. Reducing demand significantly impacts the focus on environmental and governance level sustainability in the developing countries and prioritizes survival [116]. Furthermore, amid yarn price increases due to lockdowns [117] and severe declines in consumption and employment [118], Sri Lankan factory workers had to stay home without wages and employments. These further accelerates the moving the focus away from environmental and governance level sustainability [119]. Another significant turning point of commitment from environmental and governance level sustainability is the severe brain drain in the country, losing skills, leadership, and knowledge in the firms [120]. This also make it challenging to be innovative within firms to achieve environmental and governance level sustainability [121]. Another important factor is the vast initial cost required in implementing environmental sustainability [122, 123]. Significant cost increases related to remote working methods have also constrained the budget for environmental practices and governance level sustainability implementation incentives [124] with the COVID-19.

## Managerial implications

Comparatively, small and rapidly increasing number of companies are integrating environmental and social sustainability practices in their day-to-day operations. However, integrating sustainability in multiple aspects into business operations enhances the performance of the companies, even during a period facing the risks of survival, both during the COVID-19 and the economic crisis. Therefore, sustainability can be secured the apparel firms’ survival and the future. Specially, the integration of social, ethical, and economical sustainability practices into business strategies are important to achieve an outstanding performance beyond the hardships generated by the pandemic and the recession. Interestingly, both environmental and corporate governance policies contributing to achieve sustainability may hinder the organization’s performance and firms incurring opportunity cost under the said circumstances. Therefore, temporary shifts in organization’s priorities and decision making by the managers on the above discussed significant factors are mandatory. This kind of an approach can prioritize organization’s resource allocations on environmental and governance aspects in sustainability. Moreover, a better financial stability is possible by prioritising allocation of finances. For this, temporarily cutting down massive costs can be spent on prioritised activities, such as ESG aspects and allocating the rest on aspects with the least priority. Accordingly, those firms that operates with economic, social and ethical practices can achieve competitive advantage over others than those still prioritizing on traditional environmental and governance practices during the disaster period.

## Conclusion

Since 2019, with the continuous lockdowns imposed occasionally by the government due to the pandemic and consequent economic setback, the Sri Lankan economy weakened, resulting in a spiralling economic crisis. This situation generated high inflation, which adversely affected almost all industries countrywide, repealing the economic progress achieved this far. Since the pandemic situation also affected the Sri Lankan context amid the struggle for business survival, less attention was given to sustainability, although it is one of the topical themes among scholars. In Sri Lanka, apparel and textile industry is considered the most dynamic industry because of its higher GDP contribution to the economy when compared to other industries. Most past scholars have not focused on multi-dimensions under the Sri Lankan context, except the triple bottom line encompassing economic, environment and social aspects. With the existing research gaps concerning the sustainability concept during the pandemic and economic crisis in Sri Lanka, the current study was expected to fill the knowledge gap regarding the impact of CSR practices on organizational performance in the period covered by the current study. Here, the study identifies CSR as a multi-dimensional variable, including the sub-variables economic vigour, social equity, environmental performance, ethical practices, and CG practices, focusing beyond the triple bottom line in the developing country perspective.

The study contributes positively to individual apparel firms, the entire apparel industry, the country’s economy, and scholars for future research. Doing so provides a holistic picture of how individual dimensions impact overall organizational performance rather than limiting the latter to single or three dimensions of sustainability. Further, the study findings can equip managers of apparel corporations with a better understanding of sustainability impacts on the firm’s bottom line performance. This way, managers will be well prepared to devise much-focused sustainable strategies, by considering more aspects of sustainability from a wider perspective than merely limiting to triple bottom line. Hence, when making investment decision, foreign investors place great weight on firms that have embraced and practicing the sustainability concept. This way, improving the business confidence of international investors can attract more investments in Sri Lanka. It involves overcoming the current economic crisis and putting in place sustainable strategies to create opportunities. Also, boosting foreign investments can relieve the country’s USD shortage issue by generating better organizational performance improving export income while attracting investors. This research will further enhance the existing literature by presenting data on how sustainable practices influence organizational performance in developing nations under multi-dimensional aspects, beyond the common framework of sustainability. Additionally, by adding more empirical findings on sustainability, the study will enrich the theoretically justified literature.

The study results have indicated three latent variables; economic vigour, social equity and ethical practices have significant impact on organizational performance with greater t-statistic value than (1.96) and lower p-value than (0.05). Conversely, two latent variables; environmental performance and corporate governance results demonstrated insignificant impact on organizational performance. All five variables have a positive relationship with organizational performance with positive beta values indicated in path coefficient.

Economic vigour as a latent variable of the study, indicates a significant impact on organizational performance during the pandemic and economic crisis in Sri Lanka. Here, the current study recommends apparel firms allocate more resources and pay attention to economic practices, recycling, innovative remanufacturing, the latest technological implications, using online platforms for value chain activities including purchases and sales, entering new markets, and investing in research and development. The hypothesis (H3) regarding social equity too indicated a significant impact on organizational performance. Hence, corporations can be recommended to use the latest manufacturing techniques, invest in research and development to generate innovations, use electronic media to buy and sell, enter new markets, and implement recycling, as depicted in the indicator table (Table 1) due to the significant impact indicated even during the harsh economic conditions. The latent variable, ethical practices, also demonstrate a significant impact on organizational performance, meaning that corporates have to allocate more of their resources to ensure ethical conduct within the organization during the said crisis. The study recommends companies to focus on the code of ethics, system to safeguard whistle-blowers, strategic planning, ethics training committee and evaluating role of organizational ethics, as in the indicator list. Other than these, the social dimension also indicated a significant positive impact on the organizational performance even during the pandemic and economic crisis, therefore it is better to consider about training and personal development processes, employee working conditions, health, and safety, healthcare coverage for employees, job opportunities for disabled employees and action plan to improve stakeholder involvement, as per the sustainability practices included in the Table 1.

CG and the environmental performance variables have an insignificant impact on organizational performance. Considering the insignificant impact during the period covered by the current study, corporations can reallocate resources allocated towards CG and environmental practices on other latent variables for business survival and to achieve better organizational performance. Therefore, apparel companies should less worry about practices like independent representation of directors, environmental leadership, board-level sustainability committee, transparency, CG education, life cycle analysis in product designing, renewable energy sources, lean manufacturing, reuse of water, eco-friendly venders, collaborative planning, technology for transportation, ecological friendly packaging during the pandemic and economic crisis in the country. For example, with inflation, people have other priorities and are not willing to pay higher prices for green packaging and return on investment of renewable energy sources are low in times of economic downfall. Although, recommending companies to totally ignore those dimensions is unwise, as deeper insights on the insignificant positive impact on organizational performance are pending.

This study has several limitations which can be addressed in future investigations. By analysing the combined effects of firms of varying sizes, this study does not consider the moderating effect of company size as large and SME. When considered separately, distinctive patterns can be unveiled among those two categories. Despite multiple bottom-line approaches, this study was limited to 28 sustainability practices. However, additional sustainability practices in each dimension should be included in future works because the current study results have indicated insignificant impact in environment and CG on organizational performances. The R square value of the study shows, 55.6% effect on organizational performance represented by the five observed variables. The rest of the effect (45.6%) on organization performance was represented by other unobserved variables; this is another area which the future researchers can investigate based on the current study’s limitations. Moreover, to generate better generalization and practical application, the study suggests future scholars to engage in qualitative methods to identity applicability and dig deeper into insignificant results in the harsh economic conditions.

Furthermore, this research may be extended to other developed and developing countries as the latest empirically tested sustainability model with five dimensions concerning the organizational performance of apparel firms and others. This study was limited to cross-sectional data during the COVID-19 outbreak and the subsequent economic crisis in Sri Lanka. Future researchers can get more insights from longitudinal data by comparing pre-pandemic and crisis impacts on the association between corporate sustainability practices and organizational performance. Furthermore, in addition to the apparel and textile industry, other sectors could be investigated in future studies, thereby eliminating another drawback of this research. Finally, the validity and reliability of the survey’s findings that rely on the survey questionnaire respondents’ feedback, is another disadvantage of this study. Addressing this issue in future studies enables for generalizability of findings.

## Supporting information

S1 Fig Literature source flow diagram. (TIF)

S2 Fig Research design. (TIF)
