# Supplementary figures and images for: Sustainability practices and organizational performance during the COVID-19 pandemic and economic crisis: A case of apparel and textile industry in Sri Lanka
Source: PLoS One. 2023 Jul 11;18(7):e0288179. doi: 10.1371/journal.pone.0288179 (PMC10337776; doi:10.1371/journal.pone.0288179)

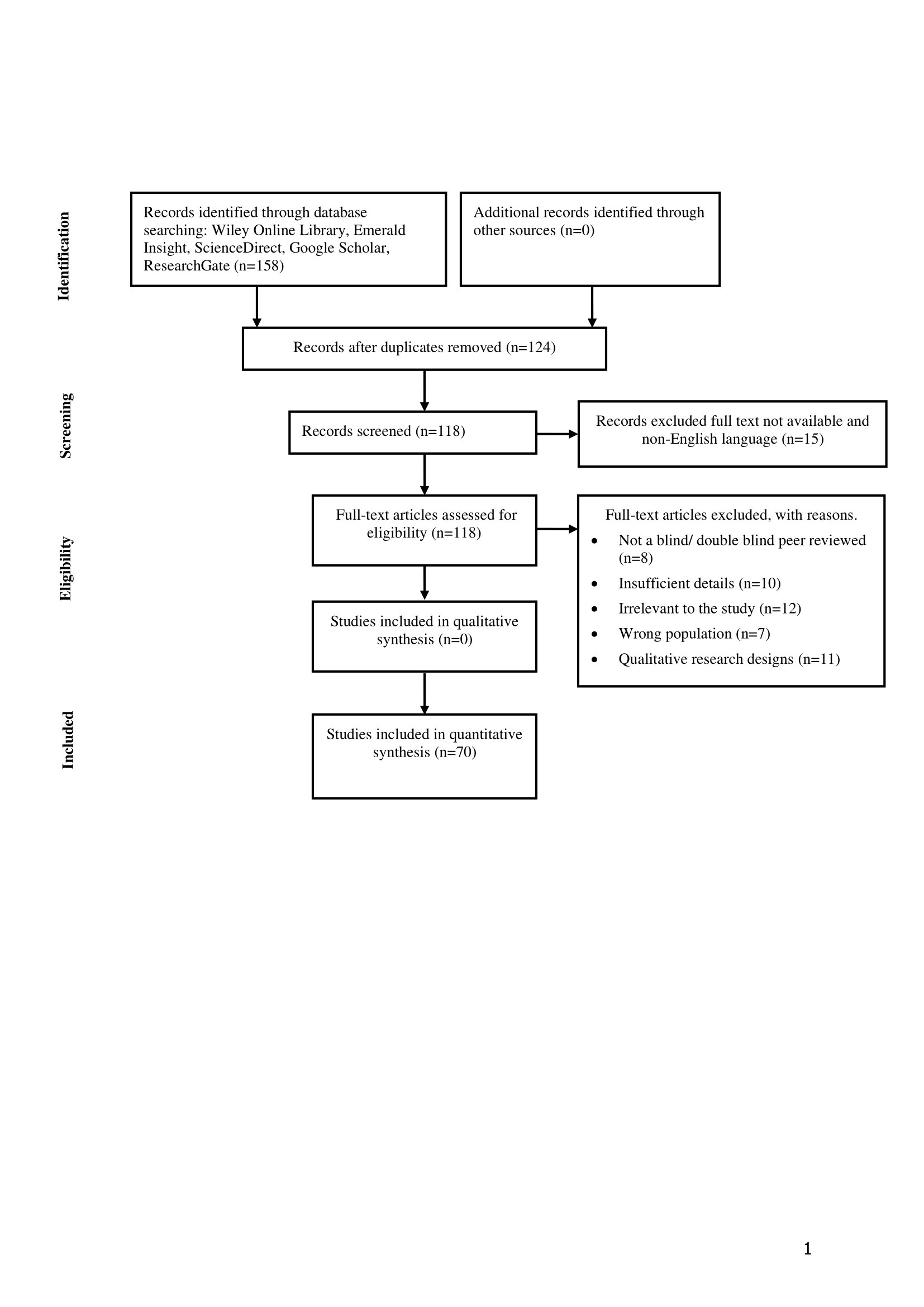

Supplement: S1 Fig — (TIF) [file pone.0288179.s001.tif]

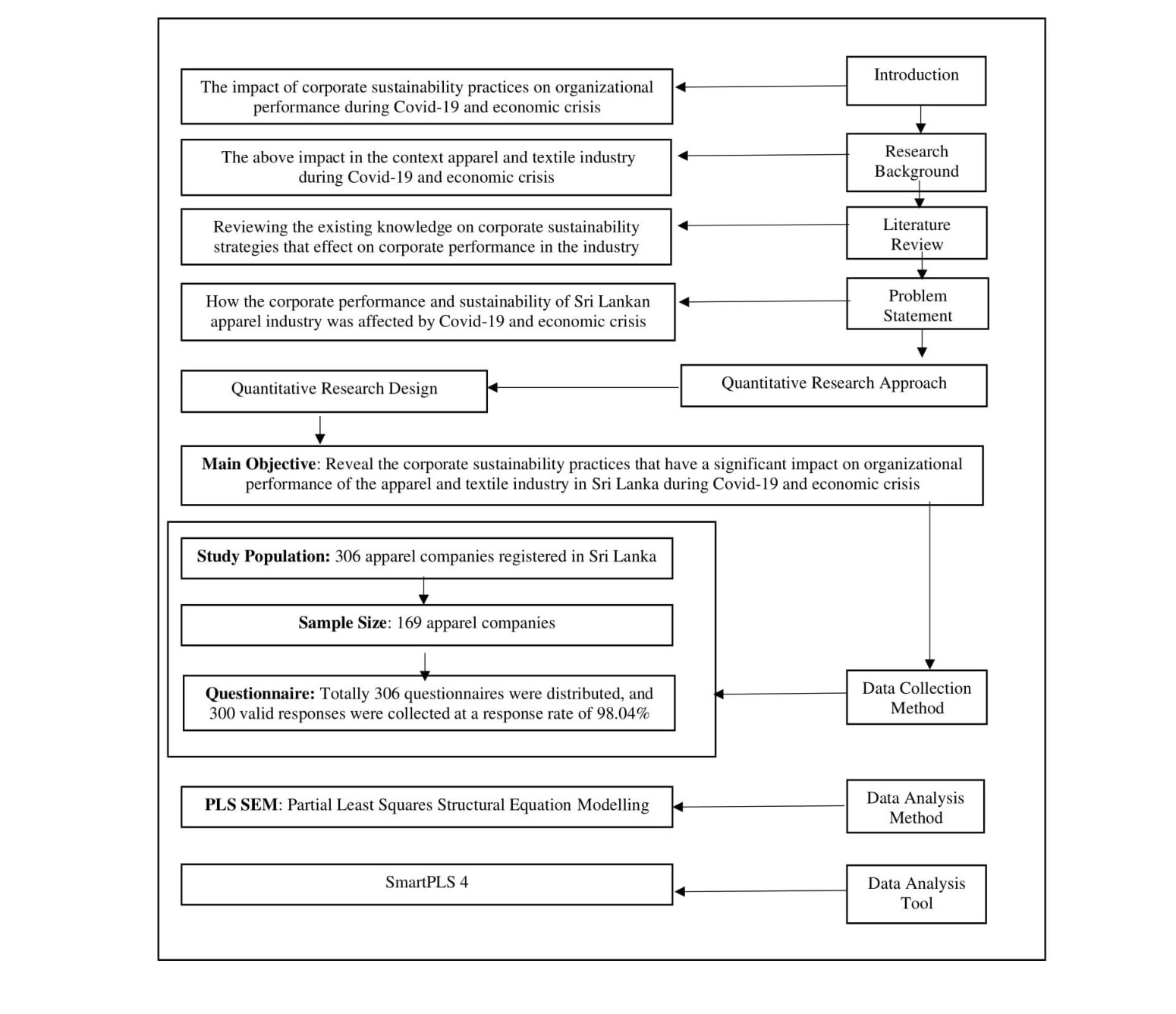

Supplement: S2 Fig — (TIF) [file pone.0288179.s002.tif]
